# The Poplar Hospital

**Published:** 1893-10-28

**Authors:** 


### The Institutional Workshop.

### WITHIN THE HOSPITALS.

### THE POPLAR HOSPITAL.

By Our Special Commissioner.

Very many of our readers will be unfamiliar with Poplar and its neighbourhood. Volumes have been written and talked of late years of the East-end of London, and Poplar contains much that has won the sympathy of the West for the East. Anyone travelling to Poplar for the first time, and making his way through the streets to the hospital, must be struck with the poverty and unattractiveness of the surroundings which confront him. The rough work of great cities has to be done, and those upon whom it devolves have necessarily to bear a heavy burden, and to live under conditions which the majority would avoid if they had any choice in the matter. Hence sympathy should evoke support for these toilers from all who are able to enjoy residence in London under the most favourable conditions. Passing along the streets the visitor cannot fail to realise all we have here written. If he is bound for the hospital, he naturally goes on to consider the added strain which such surroundings and circumstances must bring to those who work within the hospital itself. Thus we entered the Poplar Hospital with a deep feeling of sympathy for the workers within. The afternoon was dull, and outside the hospital everything tended to chill and depress, but once inside the walls these feelings gave place to a sense of gratitude and cheerfulness. Why? Because under the chairmanship of the Hon. Sydney Holland the Poplar Hospital has become one of the most inspiriting and joyous places in all London. There is no festival dinner given in aid of any object in the Metropolis throughout the year which is so helpful and beneficial to those who attend it as the Poplar dinner. Whether it be the hard conditions under which the work has to be done, or the increased sympathy which the relatively heavy trials of the patients of this particular institution evoke in those who minister to their needs, we cannot say, but the fact remains that cheerfulness, contentment, and joy prevail everywhere, and are reflected in all who labour for this hospital, or who join together to help it on its way. Would that the discontented rich, the orchids in comfortable circumstances, who have attained middle age, or the young spendthrift of unlimited means, could be induced to go to the Poplar Hospital and spend two hours in the wards under the guidance of the Matron or the Chairman of the Committee. Such a visit would do everyone of them more good than all the physic of all the doctors. The Hon. Sydney Holland would be the first, we are confident, to endorse all we have here written. His example, and the fruits which have followed his work are so encouraging as to lead us to hope that many of his class will be moved to take an interest in the voluntary hospitals, and to give personal service, as he has done, rather than money.

#### The Work of the Hospital.

Let us now consider the work which this hospital does. It can be put in a sentence. Three new accident cases are treated in every one of the twelve hours of every working day throughout the year. It is essentially an accident hospital, and as such should especially appeal to the sympathy and win the support of able-bodied citizens of all classes. It illustrates the disadvantages a nurse may suffer from who is trained at a large hospital where there is a medical school. Most nurses so trained, who believe they have thoroughly mastered their craft, find the work of the Poplar Hospital more than they can manage. Three nurses to a ward of twenty beds, and the arrival therein, as on the day of our visit, of five accident cases, all necessitating operations, and that within two and a half hours, give a fair idea of the work at Poplar. To get through it the nurses must be specially skilled in handling surgical cases, and three months in the surgical wards of a large hospital where there are many students to do the bandaging and to care for the patients generally, does not result in fitting a nurse for the services required of her at this institution.

The new wards are excellently proportioned, admirably ventilated, and thoroughly warmed and lighted. They contain most, if not all, modern appliances, including bedsteads of a special pattern, which we believe to be the best yet invented for surgical wards. The bed-heads are so constructed that they can easily be turned down to enable chloroform to be given *in loco*. The spring mattresses are planned so that fracture beds can be placed over them without the smallest difficulty. These fracture beds are divided in the middle, and are designed to combine ample strength with portability. The dressing trays, with their glass shelves, iron framework, and rubber wheels are admirable. The invalid couches; the large ward tables, fitted with cupboards to hold every kind of stores, the patients' lockers, and the bed rests have all been specially designed, and testify to the intelligent interest which has been expended upon the furniture and fittings. The baths, slop-sinks, housemaid-sinks, milk safes, and various other appliances leave nothing to be desired. Indeed, had the architect but painted the various supply pipes in different colours so as to enable each to be easily identified, and had he fitted the supply cistern to the slop sinks, so as to obviate the possibility of frequently displacing the syphons, the new wards and their appurtenances at Poplar would be almost uniquely perfect. No doubt these points will be promptly remedied, as well as the omission to provide through ventilation in the larder adjacent to the kitchen. So true is it that several of the appliances are not only excellent, but novel, that we propose to give a description of them with blocks on another occasion.

#### A Word to the Wise.

We were so fortunate as to be taken over the hospital by the Sister in charge, Miss Vacher, a most intelligent cicerone, who has evidently made a study of hospital management. Her enthusiasm, like that of the chairman, is infectious, and her thorough mastery of every detail cannot fail to impress the most indifferent visitor. Miss Vacher, we are confident, has been a tower of strength to the Poplar Hospital. Yet, strange to say, her name does not appear on the page of the report which contains the names of the committee and officers.

Miss Vacher requires a harmonium for each of the three wards, and we shall be glad to hear that amongst our readers there are three people at least who will each give £13 10s., and so provide these means of cheering the patients, who are for the most part confined to their beds. Flowers are also required, and herbaceous plants in pots. Surely hundreds of our readers have it in their power to send a supply of such things to the Poplar Hospital. Will they do so? If they hesitate, let them take the train to Poplar, interview Miss Vacher, and then the result will not be doubtful.

We commend to hospital managers generally the Poplar Hospital tripod—a simple invention, which enables the patients with damaged limbs to walk on polished floors without any risk of slipping or stumblings. We do not go further into the work of this hospital to-day, because it is still in the hands of the builder and architect Mr. Rowland Plumbe, who will not be away until March, 1894. The Hon. Sydney Holland and his friends have raised over £20,000 towards the building fund. They require another £3,000 before the end of the present year. There are, we know, many men of means who would be glad to give to a work like that which we have been describing did they know of and understand it. We would urge some of the wealthier of our readers to spend an afternoon at the Poplar Hospital, to visit its wards without notice, and to judge for themselves whether we have exaggerated anything we have here stated. We are confident that if we could induce a few of them to adopt this course the £3,000 would be gladly given in aid of a work which is unsurpassed in its excellence by any of the many splendid voluntary enterprises which tend to make this England of ours the glory and wonder of the world.

